# Multi-functional role of apolipoprotein E in neurodegenerative diseases

**DOI:** 10.3389/fnagi.2025.1535280

**Published:** 2025-01-29

**Authors:** Sadequl Islam, Arshad Noorani, Yang Sun, Makoto Michikawa, Kun Zou

**Affiliations:** ^1^Department of Neuro-Oncology, Graduate School of Medical Sciences, Institute of Brain Science, Nagoya City University, Nagoya, Japan; ^2^Department of Medicinal Chemistry, University of Kansas, Lawrence, KS, United States; ^3^Department of Geriatric Medicine, School of Life Dentistry at Niigata, The Nippon Dental University, Niigata, Japan

**Keywords:** apolipoprotein E, Alzheimer’s disease, amyloid-*β* (A*β*), Parkinson’s disease, amyotrophic lateral sclerosis, neurodegenerative diseases

## Abstract

Genetic diversity in the apolipoprotein E (ApoE) gene has been identified as the major susceptibility genetic risk factor for sporadic Alzheimer’s disease (SAD). Specifically, the *ApoEε4* allele is a significant risk factor for SAD, while *ApoEε2* allele provides protection compared to the more common *ApoEε3* allele. This review discusses the role of the ApoE in AD and other neurodegenerative disorders. ApoE, a cholesterol transport protein, influences several pathways involved in neurodegeneration, particularly in AD. Beyond its established role in amyloid *β*-protein (Aβ) metabolism and deposition, ApoE also impacts tau pathology, neurodegeneration, and the microglial response to AD. The review aims to provide an updated overview of ApoE’s diverse roles, emphasizing its involvement in Aβ clearance through ApoE receptors. It also covers ApoE’s influence in other neurodegenerative diseases like Parkinson’s disease (PD), amyotrophic lateral sclerosis (ALS), frontotemporal lobar degeneration (FTLD), Huntington’s disease (HD), vascular dementia (VD), and multiple sclerosis (MS). New research highlights the interaction between ApoE and presenilin (PS), suggesting connections between familial AD (FAD) and SAD. The review also explores protective effects of ApoE mutations against AD and ApoE4-induced tauopathy, neurodegeneration, and neuroinflammation. The insights from this comprehensive update could indeed lead to new therapeutic strategies for neurodegenerative diseases.

## 1 Introduction

Apolipoprotein E (ApoE) is a key genetic risk factor in the complex landscape of Alzheimer’s disease (AD), especially in its late-onset sporadic form. Genetic variation in the ApoE gene is a significant risk factor for late-onset AD, with the three primary ApoE allelic variants—*ApoEε2*, *ApoEε3*, and *ApoEε4*—being involved in about 95% of late-onset AD cases (Serrano-Pozo et al., 2021; Yamazaki et al., 2019). In late-onset AD, which occurs sporadically after the age of 65, the impairment of amyloid *β*-protein (Aβ) clearance mechanisms seems to be a primary factor in the accumulation of Aβ in the brain (Mawuenyega et al., 2010). Strong evidence from clinical and basic research indicates that among the three major ApoE allelic variants, the *ApoEε4* allele is associated with an increased risk of AD (Corder et al., 1993; Saunders et al., 1993) while the *ApoEε2* allele is linked to a decreased risk (Farrer et al., 1997; Corder et al., 1994) compared to the more common *ApoEε3* allele (Bu, 2009; Liu et al., 2013). Clear evidence has shown that human ApoE2 knock-in (KI) mice exhibit a significant reduction in Aβ deposition compared to ApoE3-KI and ApoE4-KI mice (Holtzman et al., 2000; Li, 2019; Fagan et al., 2002). Additionally, ApoE2 has been found to lower Aβ oligomer levels more effectively than ApoE3 and ApoE4 (Hashimoto et al., 2012). Moreover, ApoE4 has a stronger stabilizing effect on Aβ oligomers than ApoE3 (Cerf et al., 2011). Conclusive evidence shows that ApoE4 contributes to the development of AD, likely through both a loss-of-function in neuroprotection and a gain-of-function in neurotoxicity, when compared to ApoE3 (Huang and Mucke, 2012; Kanekiyo et al., 2014). For instance, homocysteine, a known risk factor for AD, prevents ApoE3 from forming dimers and reduces its ability to produce HDL. This suggests that homocysteine impairs the clearance of amyloid-beta (Aβ) by affecting the function of ApoE3 (Minagawa et al., 2010). Individuals with the *ApoEε4* allele of the ApoE gene not only have a higher risk of AD in a dose-dependent manner but also tend to experience an earlier age of onset (Liu et al., 2013; Kanekiyo et al., 2014; Sando et al., 2008). Indisputable evidence indicates that heterozygous carriers of the *ApoEε4* allele have a 3–4-fold increased risk of developing late-onset AD, while homozygous carriers face a 9–15-fold higher risk (Farrer et al., 1997; Neu et al., 2017; Genin et al., 2011). The *ApoEε2* allele reduces the risk of AD by approximately 0.6-fold (Corder et al., 1993; Holtzman et al., 2012). Among individuals with AD, the presence of *ApoEε4* is also linked to an earlier age of disease onset (Corder et al., 1993; Sando et al., 2008). A meta-analysis reported an odds ratio (OR) of 0.621 for developing AD in individuals with one *ApoEε2* allele and 3.68 for those with one *ApoEε4* allele, compared to individuals homozygous for *ApoEε3*. In cognitively healthy individuals, the allele frequencies of *ApoEε2*, *ApoEε3*, and *ApoEε4* are 7, 79, and 14%, respectively, whereas in AD patients, the frequencies are 4, 58, and 38%. These findings highlight that *ApoEε4* significantly increases the risk of AD and lowers the age of onset in a dose-dependent manner (Yamazaki et al., 2019). Several *in vitro* and *in vivo* studies suggest that the ApoE4 protein isoform promotes amyloid pathology and disrupts various aspects of normal brain function, indicating a gain-of-function effect in neurotoxicity associated with ApoE4 (Liu et al., 2013). While the distinct effects of ApoE isoforms on amyloid pathology and Aβ metabolism have been confirmed at molecular, cellular, and organismal levels, the precise mechanisms remain not fully understood, and proposed mechanisms continue to be debated. Recent studies, however, have brought attention to pathways not dependent on Aβ that are differentially influenced by ApoE, such as tau-driven neurodegeneration (Shi et al., 2017), microglial responses, and the risks associated with dementia with Lewy bodies (DLB) (Bras et al., 2014; Tsuang et al., 2013; Guerreiro et al., 2018), Parkinson’s disease dementia (PDD) (Tsuang et al., 2013; Huang et al., 2006; Irwin et al., 2012; Tropea et al., 2018) and the extent of TAR DNA-binding protein 43 (TDP-43) pathology in AD (Josephs et al., 2014; Wennberg et al., 2018; Yang et al., 2018).

**Figure 1:**
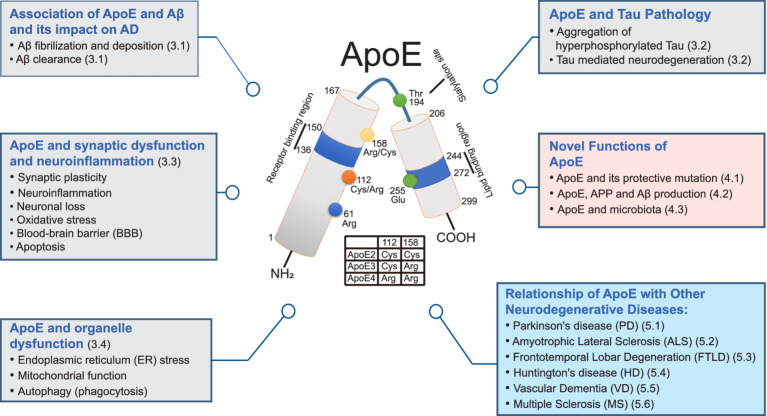
Multifaceted roles of ApoE in neurodegenerative disorders: a comprehensive diagram. This diagram illustrates the diverse functions of ApoE in Alzheimer’s disease (AD) and other neurodegenerative disorders. Grey boxes represent functions associated with ApoE contributing to AD through Aβ fibrilization and deposition, Aβ clearance, aggregation of hyperphosphorylated tau, neuron loss, and organelle dysfunction. Pink boxes highlight novel functions of ApoE, indicating potential roles of ApoE and related mechanisms. Blue boxes depict the relationships between ApoE and other degenerative diseases. In the center, the illustration of structural and functional regions of ApoE are displayed. Human ApoE is a heavily glycosylated protein with 299 amino acid residues containing a receptor-binding region (residues 136–150) in the N-terminal domain (residues 1–167) and a lipid-binding region (residues 244–272) in the C-terminal domain (residues 206–299). Human ApoE exists three isoforms the ApoE2, ApoE3 and ApoE4 which are distinguished by the amino acid residues located at residues 112 and 158. ApoE2 isoform has Cys residues at both positions, ApoE3 isoform has a Cys residue at 112 and an Arg residue at 158, and apoE4 has Arg residues at both positions.

In this review, we aim to explore the emerging connections between ApoE genotype and pathogenic proteins in individuals with AD and other neurodegenerative disorders. Moving beyond traditional viewpoints, we shed light on the multifaceted role of ApoE in its interaction with Aβ, elucidating the involvement of ApoE receptors in the clearance of Aβ. Our review entails the latest research findings on ApoE’s functions and its impact on AD, Parkinson’s disease (PD), amyotrophic lateral sclerosis (ALS), frontotemporal lobar degeneration (FTLD), Huntington’s disease (HD), vascular dementia (VD), and multiple sclerosis (MS), thus providing a fresh and insightful perspective on unraveling the mechanisms underlying neurodegenerative diseases. Additionally, we review newly discovered ApoE functions and their close relationships with known cellular physiology and pathological processes. Through a comprehensive analysis of findings from multiple studies, this review underscores the multifaceted roles of ApoE and their implications in AD and other neurodegenerative conditions (Figure 1).

## 2 Biology of ApoE

### 2.1 Structure

Human ApoE is a ~ 34 kDa glycoprotein composed of 299 amino acids and is part of the family of amphiphilic exchangeable lipoproteins (Mahley, 1988). The human ApoE gene is situated on chromosome 19q13.32 and consists of four exons and three introns (Das et al., 1985; Fernández-Calle et al., 2022). There are three primary ApoE alleles in humans: *ApoEε2*, *ApoEε3*, and *ApoEε4*. These alleles were first identified through the characterization of human ApoE polymorphisms using isoelectric focusing of plasma samples from patients with familial lipoprotein disorder type III hyperlipoproteinemia (Kanekiyo et al., 2014; Utermann et al., 1977). The ApoE isoforms encoded by the three gene alleles display distinct band patterns with specific isoelectric points: ApoE2 (pH 5.4), ApoE3 (pH 5.55), and ApoE4 (pH 6.1) (Utermann et al., 1977; Mahley and Rall, 2000). Studies indicate that the variability among the three major ApoE isoforms—ApoE2, ApoE3, and ApoE4—is attributable to genetic polymorphisms (Zannis and Breslow, 1981). Recent review studies have documented that human ApoE genotypes can be classified into six major types based on allelic variations: three homozygous genotypes (ApoE2/2, ApoE3/3, and ApoE4/4) and three heterozygous genotypes (ApoE2/3, ApoE2/4, and ApoE3/4) (Yamazaki et al., 2019; Chen et al., 2021). Complete amino acid sequencing has shown that the three ApoE gene alleles differ only at positions 112 and 158: ApoE2 has Cys112 and Cys158; ApoE3 has Cys112 and Arg158; and ApoE4 has Arg112 and Arg158 (Figure 1) (Rall et al., 1982; Weisgraber et al., 1981). However, these single amino acid polymorphisms significantly affect ApoE functionality, leading to isoform-specific variations in structure that influence its binding properties with lipids and receptors, as well as its propensity for oligomerization and stability (Yamazaki et al., 2019; Ruiz et al., 2005; Yamazaki et al., 2016; Hatters et al., 2006; Ma et al., 2018; Raulin et al., 2022). Therefore, understanding the differences between ApoE isoforms is crucial for elucidating its function.

The Nuclear Magnetic Resonance (NMR)-based model created from a monomeric mutant form of ApoE3, which has mutations in the C-terminal domain to prevent aggregation, provided the first complete view of ApoE’s full-length structure. However, the precise structure of native ApoE remains uncertain due to the protein’s tendency to aggregate (Chen et al., 2011). The human ApoE protein comprises two primary structural domains: an N-terminal domain and a C-terminal domain, connected by a flexible hinge region (Chen et al., 2011). The N-terminal domain (residues 1–167) includes the receptor-binding region (Wilson et al., 1991), while the C-terminal domain (residues 206–299) contains the lipid-binding region (Weisgraber, 1994). These domains are separated by the hinge region (residues 168–205) (Chen et al., 2011). X-ray crystallographic analyses (Wilson et al., 1991) and NMR-based working models (Sivashanmugam and Wang, 2009) have identified a four-helix bundle in the N-terminal domain of ApoE. The structure of the N-terminal domain in a monomeric mutant form of ApoE3 shows that Arg 61 forms a hydrogen bond with Thr 194, and Glu 255 forms a salt bridge with Lys 95 (Kanekiyo et al., 2014; Chen et al., 2011). In contrast, the N-terminal domain structure of ApoE4 shows that Arg 112 forms a salt bridge with Glu 109, causing Arg 61 to be exposed away from the four-helix bundle. In ApoE3, however, this side chain is buried, as revealed by X-ray crystallographic analyses (Dong et al., 1994). Consequently, it is predicted that Arg 61 in ApoE4 interacts with Glu 255, facilitating interactions between the N- and C-terminal domains (Mahley and Rall, 2000; Wilson et al., 1991; Mahley et al., 2009). Domain interaction is a unique structural characteristic of ApoE4 that sets it apart from ApoE2 and ApoE3. Unlike ApoE4, ApoE2 and ApoE3 lack domain interaction due to the presence of Cys-112 instead of Arg-112 (Raffai et al., 2001). The unique Cys-158 residue in ApoE2 disrupts the salt bridge between Arg-158 and Asp-154 and forms a new salt bridge between Arg-150 and Asp-154, leading to reduced affinity for the low-density lipoprotein receptor (LDLR) (Dong et al., 1994). This diminished binding to LDLR places ApoE2 homozygous individuals at a greater risk for type III hyperlipoproteinemia, a genetic disorder characterized by elevated plasma levels of cholesterol and triglycerides (Hatters et al., 2006). Although the NMR structure does not show domain-domain interactions in ApoE4, fluorescence resonance energy transfer (FRET) and electron paramagnetic resonance (EPR) analyses have indicated that such interactions are stronger in ApoE4 compared to ApoE3. Studies have also shown that the distance between Arg 61 and Glu 255 is closer in both lipid-free and phospholipid-bound ApoE4 (Hatters et al., 2005). Similarly, ApoE4-expressing neuronal cells also showed stronger domain-domain interaction by live cell imaging (Xu et al., 2004). Beyond these interactions, the single amino acid difference at position 112—Cys in ApoE3 and Arg in ApoE4—has a significant impact on the structure and functions of ApoE, particularly concerning AD pathways.

### 2.2 ApoE production, lipidation, and impact of ApoE isoform on receptor binding

ApoE production and secretion are highly specific to particular cells and tissues (Fernández-Calle et al., 2022; Kockx et al., 2018). ApoE is primarily produced by hepatocytes and macrophages in the liver (Huang et al., 2004). Although ApoE does not cross the blood–brain barrier (BBB), it is abundantly expressed in the central nervous system (CNS) by astrocytes, microglia, vascular mural cells, and choroid plexus cells, and to a lesser extent in stressed neurons (Boyles et al., 1985; Xu et al., 2006; Aoki et al., 2003; Mahley and Huang, 2012a; Grubman et al., 2019; Kang et al., 2018). Furthermore, various cell types within the BBB, such as oligodendrocytes and pericytes can also produce ApoE to a lesser degree (Fernández-Calle et al., 2022; Bruinsma et al., 2010). In both astrocytes and microglia, ApoE2 is secreted to a greater extent than ApoE4 into the culture medium (Hasel and Liddelow, 2021). In primary cultures from human ApoE-targeted replacement (ApoE-TR) mice, astrocytes secreted at least six times more ApoE than microglia under basal conditions. ApoE2 astrocytes released more ApoE than ApoE4 astrocytes, though cellular ApoE levels were consistent across genotypes. Approximately 50–60% of total ApoE produced by astrocytes was secreted, regardless of genotype. While ApoE mRNA levels showed no significant differences among genotypes, ApoE4 astrocytes displayed a trend toward higher ApoE mRNA than ApoE2. In microglia, secreted ApoE2 was significantly higher than ApoE3 and ApoE4, and cellular ApoE levels were also greater in ApoE2 than in ApoE3 and ApoE4. The proportion of secreted ApoE relative to total ApoE in microglia was highest in ApoE2 (49%) and lowest in ApoE4 (30%). Notably, ApoE4 secretion was reduced in microglia (30%) compared to astrocytes (50%) (Lanfranco et al., 2021).

**Figure 2:**
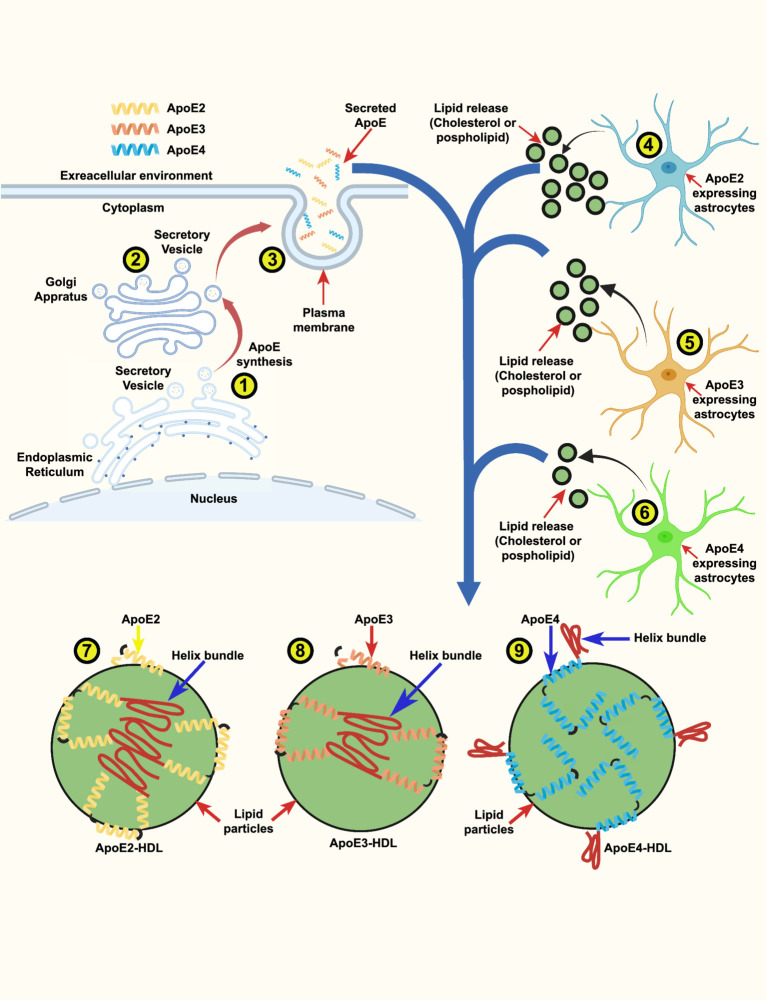
ApoE isoform-dependent lipid release from astrocytes and generation of ApoE-HDL particles: a schematic illustration. ApoE is vesicular protein that is produced and secreted through a classical secretory pathway. ApoE is synthesized in the endoplasmic reticulum (ER) (1), after which it undergoes post-translational modification in the Golgi apparatus (2). Subsequently, it is transported to the plasma membrane and secreted to the extracellular environment (3). The amounts of lipid particles (cholesterol and phospholipids) release from (3) ApoE2-expressing astrocytes (4) ApoE3-expressing astrocytes and (5) from ApoE4-expressing astrocytes (6) derived from human ApoE2, ApoE3 and ApoE4 knock-in (KI) mouse brains. The release potency of lipid particles followed the rank order: ApoE2 > ApoE3 > ApoE4. To ensure proper functionality the secreted ApoE necessitates to bound to lipid particles to form ApoE-HDL. Model of ApoE lipid particles generated by ApoE2-, ApoE3- and ApoE4-expressing astrocytes. The intricate illustration indicate that ApoE2 has higher ApoE-HDL levels than ApoE3 and ApoE4 (7). ApoE3 has the ability to generate similarly sized ApoE lipid particles with a smaller number of ApoE molecules (8) than ApoE4 (9). Based on these findings, we propose a detailed sketch showing that one ApoE4-containing lipid particle contains ∼2-fold numbers of ApoE molecules (8) compared with an ApoE3-containing lipid particle (8).

ApoE is produced and secreted through a classical secretory pathway: Initially, it is synthesized in the endoplasmic reticulum (ER), after which it undergoes post-translational glycosylation and sialylation in the Golgi network. Subsequently, it is transported to the plasma membrane and secreted (Figure 2) (Kockx et al., 2018; Kockx et al., 2007). Upon secretion from cells, ApoE mediates the uptake of cholesterol and phospholipids via interactions with transmembrane ATP-binding cassette (ABC) transporters, particularly ABCA1 and ABCG1, culminating in the assembly of lipoprotein particles (Hirsch-Reinshagen et al., 2004; Wahrle et al., 2004; Karten et al., 2006). ABCA1 and ABCG1 are predominantly expressed in neurons, glial cells, and macrophages, serving to facilitate the efflux of cholesterol to ApoE (Karasinska et al., 2013; Vance and Hayashi, 2010; Tarr and Edwards, 2008). The pivotal role of ApoE lies in the redistribution of cholesterol and phospholipids to neurons through its interaction with cell-surface ApoE receptors. Hence, to ensure proper functionality, ApoE necessitates both secretion and lipidation (Fernández-Calle et al., 2022). Additionally, prior studies have indicated that ApoE3 facilitates the efflux of cholesterol and phospholipids from both astrocytes and neurons more effectively than ApoE4. This distinction is attributed to the intramolecular domain interaction and intermolecular dimerization of ApoE3, which augment the generation of HDL-like particles, a characteristic not observed in ApoE4 (Figure 2) (Minagawa et al., 2009; Gong et al., 2002; Michikawa et al., 2000; Gong et al., 2007).

ApoE3 and ApoE2 have a stronger attraction to high-density lipoproteins (HDL), while ApoE4 is often linked to very low-density lipoproteins (VLDL) and low-density lipoproteins (LDL) (Dong et al., 1994; Hatters et al., 2006). The ApoE protein comprises two primary functional regions: the receptor-binding site (residues 136–150) within the N-terminal domain and the lipid-binding site (residues 244–272) within the C-terminal domain. NMR analysis of ApoE3 has elucidated that exposed hydrophobic residues result in the destabilization of the C-terminal domain, leading to the formation of a large exposed hydrophobic surface that facilitates the initial binding of lipids. However, it is noteworthy that several hydrophilic residues in the C-terminal domain are situated within the domain interface (Kanekiyo et al., 2014; Hatters et al., 2006). In ApoE4, the substitution of Arg 61 with Thr or Glu 255 with Ala induces a preference for HDL like that of ApoE3 (Dong et al., 1994; Hatters et al., 2006). Hence, it is likely that Arg 61, Glu 255, or their interaction is indispensable for VLDL preference in ApoE4. This implies that the distinct lipoprotein preference of ApoE4 increases plasma cholesterol levels, consequently elevating the risk of cardiovascular diseases, particularly atherosclerosis (Kanekiyo et al., 2014; Davignon et al., 1988).

Lipidated ApoE is internalized via ApoE receptors, specifically the LDL receptor (LDLR) family, which encompasses LDLR and LDLR-related protein 1 (LRP1) receptors (Yamazaki et al., 2019; Holtzman et al., 2012; Husain et al., 2021). LDLR is a member of the LRP family, composed of seven structurally similar transmembrane proteins, including LRP1, LRP1b, LRP2, LRP4, LRP8, VLDLR, and LDLR (May et al., 2007). LRPs and VLDLR bind to all forms of ApoE particles, with VLDLR also capable of binding to lipid-free ApoE, while LDLRs exhibit a higher affinity for lipidated ApoE particles (Bu, 2009; Ruiz et al., 2005). Genetic variations in ApoE directly influence its lipidation status in an isoform-specific manner (ApoE4 < ApoE3 < ApoE2) (Hubin et al., 2019; Morrow et al., 2000). The lipidation status of ApoE is instrumental in its receptor-binding properties, molecular stability, and functional integrity. Isoform disparities of ApoE markedly alter its composition and operation, consequently impacting its interaction with both lipids and receptors. Research indicates that ApoE3 possesses an increased ability, approximately 2.5 to 3.9-fold, to induce lipid efflux compared to ApoE4 (Minagawa et al., 2009). In a previous study, it was shown that ApoE2 has a binding affinity to LDLR that is more than 50 times weaker than the binding of ApoE3 or ApoE4 to this receptor (Hatters et al., 2006). This low binding propensity of ApoE2 for LDLR leads to impaired clearance of triglyceride-rich lipoprotein remnant particles, thereby contributing to the onset of type III hyperlipoproteinemia (Mahley, 1988; Phillips, 2014). On the other hand, ApoE4 has a higher binding affinity to VLDL particles compared to ApoE2 and ApoE3 (Hatters et al., 2006), which impairs the lipolytic processing of VLDL in the periphery. Consequently, ApoE4 exacerbates pro-atherogenic changes in lipoprotein distribution (Phillips, 2014). The lipidation state of ApoE significantly affects its physiological and pathological roles, and increasing ApoE lipidation has been suggested as a target for AD treatment (Lanfranco et al., 2020).

In addition, ApoE not only interacts with the LDLR family but also with cell surface heparan sulfate proteoglycans (HSPG) (Ji et al., 1993), either in its lipid-free or lipidated forms (Saito et al., 2003). This binding occurs through both the receptor-binding region in the N-terminal domain and the basic residues around Lys233 in the C-terminal domain (Saito et al., 2003). Additionally, LRP1 can form a complex with HSPG (Ji et al., 1993). In the HSPG/LRP1 uptake pathway, ApoE either binds initially to HSPG and is subsequently transferred to LRP1 for uptake, or it binds directly to the HSPG/LRP1 complex (Ji et al., 1993). The receptor-binding functionality of ApoE is isoform-dependent. ApoE3 and ApoE4 exhibit similar binding affinity to LDLR, whereas ApoE2 has a significantly lower affinity (Schneider et al., 1981). However, the reduced binding affinity of ApoE2 to LRP1 is less pronounced compared to LDLR (Kowal et al., 1990). Interestingly, there appear to be no substantial differences among ApoE isoforms in their capacity to bind to HSPG (Mahley and Rall, 2000). Moreover, the ApoE3-R136S mutation, also known as ApoE3 Christchurch (ApoE3-Ch), has recently gained attention for its significantly reduced heparin-binding ability (Arboleda-Velasquez et al., 2019). The ApoE3-Ch mutation strongly protects against early-onset AD, as evidenced by a homozygous index patient who developed only mild dementia much later in life than expected, highlighting the importance of studying rare ApoE variants in AD pathogenesis and protection (Arboleda-Velasquez et al., 2019). This mutation occurs in a region of ApoE that is critical for binding to lipoprotein receptors and HSPG (Mahley et al., 1999). Previous analyses revealed that ApoE4 exhibits a higher affinity for heparin compared to ApoE3 and ApoE2 (Yamauchi et al., 2008). HSPG is believed to promote Aβ aggregation and facilitate the neuronal uptake of extracellular tau, with ApoE binding potentially playing a key role in these processes (Rauch et al., 2018). A monoclonal antibody (1343A) was developed to target ApoE amino acids 130–143, including the R136S mutation, and demonstrated the ability to reduce the heparin-binding capacity of wild-type (WT) ApoE3 to levels similar to ApoE3-Ch *in vitro*. These findings suggest that targeting this specific region of ApoE with antibodies or other molecules, or modulating ApoE-HSPG interactions, may mimic the potentially protective effects associated with ApoE3-Ch (Arboleda-Velasquez et al., 2019).

### 2.3 ApoE receptors in microglia and astrocytes: similarities and differences

Although the primary ApoE receptors include LDLR, LRP1, VLDLR, and ApoE receptor 2 (ApoER2), microglia and astrocytes also express a diverse collection of receptors. Microglia possess a variety of receptors, including those known as pattern recognition receptors (PRRs), which can detect stimuli of diverse origins. The key PRRs include toll-like receptors (TLRs), inflammasome-forming nucleotide-binding oligomerization domain-like receptors (NLRs), the receptor for advanced glycation end-products (RAGE), and the triggering receptor expressed on myeloid cells 2 (TREM2) (Kigerl et al., 2014; Ulland and Colonna, 2018). In the brain, TREM2 is predominantly expressed by microglia (Ulland and Colonna, 2018), making it crucial to understand its microglia-associated functions and how they are influenced by the ApoE genotype under disease conditions. Typical TREM2 ligands include phosphatidylserine found in apoptotic cells, as well as glycolipids, sphingomyelin, and sulfatide present in damaged myelin. Additionally, other identified ligands for TREM2 include ApoE, clusterin (CLU/ApoJ), Aβ, phosphatidylinositol, and phosphatidylcholine (Ulland and Colonna, 2018; Gratuze et al., 2018). Another group of studies has emphasized the critical roles of TREM2 in the seeding and spreading of both Aβ and tau pathologies (Parhizkar et al., 2019; Leyns et al., 2019). In a seeding model involving intracerebral injection of Aβ-rich brain extracts from AD patients or aged APP mice, the absence of TREM2 resulted in an increased number of seeded amyloid plaques with reduced levels of microglia-derived ApoE deposition within the plaques in mouse brains (Parhizkar et al., 2019). Consistent with these findings, reduced ApoE levels in amyloid plaques were also observed in the brains of AD patients carrying various TREM2 loss-of-function variants (Parhizkar et al., 2019).

Although TREM2 can also be expressed in astrocytes, neurons, and oligodendrocytes, its primary and most critical role is as a microglial receptor essential for maintaining neuronal health (Kober et al., 2021). *In vitro* models have demonstrated that the Axl receptor acts as a potential regulator of ApoE homeostasis in astrocytes (Gratuze et al., 2018). Axl, along with Mer and Tyro3, belongs to the TAM receptor family (Lu and Lemke, 2001; Fourgeaud et al., 2016), which has long been associated with the phagocytic clearance of apoptotic cells (Shinohara et al., 2010). In APP/PS1 mice lacking TAM receptors, the transcriptomic response to Aβ plaques was diminished, including the downregulation of genes involved in lipid metabolism, such as ApoE (Huang et al., 2021). Another receptor, LRP1, is a cell surface receptor involved in endocytosis and signal transduction. While it is expressed in microglia, it is also highly expressed in neurons and, to a lesser extent, in neuroblasts, radial glia, astrocytes, and oligodendrocyte progenitor cells (OPCs) (Auderset et al., 2016). In vitro studies using primary microglial cells have shown that LRP1 activation suppresses microglial activation by modulating the JNK and NF-κβ signaling pathways (Yang et al., 2016; Chuang et al., 2016). Additionally, LRP1 is thought to mediate ApoE’s effects on microglial inflammation (Pocivavsek et al., 2009). Microglia are also the primary source of the complement system component C1q. It has been demonstrated that all ApoE isoforms inhibit the classical complement cascade by binding to C1q with high affinity, thereby interfering with the early activation stages. The formation of C1q-ApoE complexes has been associated with cognitive decline, and suppression of complement protein C5 has been shown to reduce inflammation (Yin et al., 2019).

## 3 ApoE in Alzheimer’s disease

In humans, it has been found that the *ApoE ε3* allele is the most common (77%), while the *ApoEε2* allele is the least common (8%) (Mahley, 1988). There is strong evidence indicating that the *ApoEε4* allele significantly increases the risk of AD (Yamazaki et al., 2019; Farrer et al., 1997) and decreases the age of disease onset (Corder et al., 1993; Sando et al., 2008) in a manner that depends on the number of alleles. The *ApoEε4* allele is the most prominent genetic risk factor for AD, with an allele frequency of around 40% in AD patients compared to 15% in the general population (Farrer et al., 1997; Kanekiyo et al., 2014). The frequency of AD and the average age at clinical onset varies among ApoEε4 homozygotes (91% and 68 years of age), *ApoEε4* heterozygotes (47% and 76 years of age), and *ApoEε4* non-carriers (20% and 84 years) (Corder et al., 1993). ApoE is involved in various processes, including both Aβ-dependent and Aβ-independent pathways, which are differentially influenced by the specific ApoE isoforms. For example, the ApoE isoforms differ in their effects on Aβ accumulation and clearance. In contrast, the Aβ-independent roles of ApoE involve tau-related abnormalities, tau-driven neuronal damage, and microglial responses to AD-related conditions.

### 3.1 Roles of ApoE in Aβ deposition and Aβ clearance

The relationship between the ApoE genotype and the pathological deposition of Aβ is isoform dependent (Raulin et al., 2022). Extensive evidence illustrates the isoform-dependent co-deposition of ApoE with Aβ in amyloid plaques (ApoE4 > ApoE3 > ApoE2) (Hashimoto et al., 2012; Namba et al., 1991). Notably, studies have consistently shown that ApoE4-TR mice exhibit elevated Aβ levels in both brain tissues and cerebrospinal fluid (CSF) compared to ApoE3-TR mice (Bales et al., 2009; Holtzman et al., 2000). Furthermore, pathological analyses of post-mortem brain tissue from individuals with AD have revealed heightened intra-neuronal accumulation of Aβ in ApoE4 carriers (Christensen et al., 2010), plaque deposition in the brain parenchyma (Polvikoski et al., 1995; Schmechel et al., 1993; Tiraboschi et al., 2004), formation of neurotoxic Aβ oligomers (Koffie et al., 2012), and extensive cerebral amyloid angiopathy (CAA) (Rannikmäe et al., 2014; Shinohara et al., 2016). Individuals who carry the ApoE2 gene variant exhibit reduced amyloid plaque numbers, less severe pathology, and maintained cognitive function (Serrano-Pozo et al., 2015). In murine models, it was observed that human ApoE4 knock-in (KI) mice display increased Aβ deposition compared to human ApoE3-KI mice (Holtzman et al., 2000), while human ApoE2 expressing mice showed a marked reduction in Aβ deposition (Fagan et al., 2002). The interaction of ApoE4 with oligomers and fibrils of Aβ has been found to impact the kinetics of amyloid aggregation, indicating that ApoE4 stabilizes soluble, cytotoxic oligomeric Aβ fragments, thereby enhancing fibril formation and promoting Aβ deposition (Garai et al., 2014). Furthermore, the overexpression of ApoE4 in murine models, where ApoE4 is conditionally induced in astrocytes, has been demonstrated to expedite the initial seeding of amyloid pathology, resulting in a significant increase in plaque deposition and an extended Aβ half-life in the brain (Liu et al., 2017). During the seeding stage in amyloid precursor protein (APP)/presenilin-21 (APP/PS1-21) animal models, the inhibition of ApoE4 with antisense oligonucleotides (ASOs) results in the formation of larger Aβ plaques with reduced plaque-associated neuritic dystrophy (Huynh et al., 2017). In AD model mice overexpressing human mutant APP, the knockout of endogenous ApoE leads to a significant reduction in Aβ deposition in the brain, along with lowered Aβ40 and Aβ42 deposits, underscoring the substantial role of ApoE in Aβ fibril formation and amyloid deposition (Holtzman et al., 2000; Bales et al., 1999; Bales et al., 1997). Conversely, the complete absence of ApoE expression in AD mouse models (APP/PS1 mice) containing both APP and PS1 mutations enhances Aβ deposition and overall plaque size (Ulrich et al., 2018). Furthermore, studies have demonstrated the distinct impact of ApoE isoforms in mice. The mouse ApoE gene, situated on chromosome 7, exists as a single isoform and exhibits interactions like those of human ApoE3, with <40% similarity in the promoter regions (Raffai et al., 2001; Maloney et al., 2007). Consequently, mouse ApoE differs in functionality regarding Aβ clearance, neuroinflammation, and synaptic integrity. Notably, mouse ApoE significantly accelerates early Aβ deposition compared to human ApoE2 (and, to a lesser extent, ApoE3), indicating the protracted capacity of human ApoE2 and ApoE3 to impede Aβ conversion into fibrillar forms.(Fagan et al., 2002; Tai et al., 2017; Liao et al., 2015).

Studies have conducted an in-depth examination of the potential impact of ApoE isoforms on Aβ clearance. It has been observed that ApoE plays a crucial role in facilitating Aβ clearance in an isoform-dependent manner, with ApoE2 being more effective than ApoE3, and ApoE4 exhibiting the lowest efficiency. This facilitation is achieved through the activation of proteolytic degradation via endopeptidases such as neprilysin (NEP) and insulin-degrading enzyme (IDE) (Saido and Leissring, 2012; Russo et al., 1998; Jiang et al., 2008). ApoE also enhances Aβ clearance through the activation of cellular uptake (phagocytosis) and subsequent degradation by brain parenchymal cells (neurons, astrocytes, and microglia), with its efficiency being ApoE isoform-dependent (Kiani, 2023; Koistinaho et al., 2004; Lin et al., 2018). The cellular uptake of Aβ by astrocytes and microglia likely represents a key pathway for Aβ clearance. When brain sections containing Aβ plaques from amyloid model mice were cultured with adult mouse astrocytes, the astrocytes internalized and degraded Aβ in a manner dependent on both ApoE and LRP1(Koistinaho et al., 2004). In microglia, soluble Aβ is likely internalized via fluid-phase macropinocytosis into lysosomes for degradation (Mandrekar et al., 2009), while Aβ aggregates interact with a multicomponent cell surface receptor complex and are internalized through phagocytosis (Bamberger et al., 2003). Neurons also contribute to Aβ clearance through uptake and lysosomal degradation (Li et al., 2012). Notably, neurons are at the highest risk of encountering Aβ in the brain, as Aβ is predominantly produced by neurons. The functional role of neurons in Aβ clearance depends on LRP1 function. A previous study demonstrated that when LRP1 is deleted exclusively in neurons in the adult mouse brain, the half-life of interstitial fluid (ISF) Aβ increases, leading to greater Aβ accumulation and pathology (Kanekiyo et al., 2013). Studies have also highlighted the influence of ApoE isoforms on other clearance pathways, such as cerebrospinal fluid (CSF) absorption into the circulatory and lymphatic systems, clearance via ISF bulk flow, as well as clearance through the BBB (Kanekiyo et al., 2014; Tarasoff-Conway et al., 2015). Furthermore, research findings indicate that ApoE4-TR mice exhibit Aβ40 aggregation in the peri-arterial drainage pathway following intracerebral injections, a phenomenon not observed in ApoE3-TR mice (Hawkes et al., 2012). An *in vivo* study has shown that ApoE2 and ApoE3, along with Aβ-ApoE2 and Aβ-ApoE3 complexes, are cleared at the BBB through both VLDLR and LRP1 at a significantly faster rate than Aβ-ApoE4 complexes (Deane et al., 2008). Before binding to the receptor, ApoE necessitates the generation of high-density lipoprotein (HDL)-like particles through its association with cholesterol and phospholipids. A study has shown that astrocytes expressing ApoE3 release 2.5 times more cholesterol than those expressing ApoE4 and that ApoE3 facilitates the creation of similarly sized HDL particles with a reduced number of ApoE3 molecules compared to ApoE4 (Figure 2) (Gong et al., 2002; Michikawa et al., 2000). However, ApoE may also hinder Aβ clearance by competitively binding with Aβ for receptors or by impeding Aβ clearance through the blood–brain barrier in an isoform-dependent manner (ApoE4 > ApoE3) (Yamazaki et al., 2019; Kanekiyo et al., 2014; Kanekiyo et al., 2013). Recently, a novel association between ApoE and PS has been discovered, linking the mechanisms associated with FAD and SAD. PS constitutes the primary core component of the *γ*-secretase complex, responsible for the proteolytic processing of APP to generate a series of amyloidogenic Aβ species (Bai et al., 2015). Most mutations in the *PSEN1* and *PSEN2* genes augment the Aβ42/Aβ40 ratio, resulting in the early onset of FAD (Borchelt et al., 1996; Sun et al., 2017). We found that the absence of PS in fibroblasts leads to a complete cessation of ApoE secretion, accompanied by the nuclear translocation of ApoE from the cytosol (Islam et al., 2022). PS mutations and ApoE4 have long been considered to cause FAD and SAD by two independent mechanisms, abnormal Aβ generation and impaired Aβ clearance, respectively. Evidence suggests that PS mutations or altered γ-secretase activity play a role in Aβ clearance through the regulation of ApoE secretion. Consequently, enhancing ApoE levels by elevating PS function may augment Aβ clearance. These findings substantiate the notion that ApoE isoforms differentially govern Aβ deposition and clearance through their interaction with Aβ, indicating that the ApoE-Aβ interaction could serve as a promising target for therapeutic intervention during the early stages of the disease.

### 3.2 ApoE and tau pathology

Previous research has indicated that the presence of ApoE4 significantly intensifies tau-induced neurodegeneration in a murine model of tauopathy (Shi et al., 2017). Conversely, the homozygous ApoE3-R136S mutation has been observed to mitigate ApoE4-triggered tau pathology, neurodegeneration, and neuroinflammation (Nelson et al., 2023). The *ApoEε4* allele exerts influence on neurodegenerative conditions characterized by the presence of neurofibrillary tangles (NFTs) comprised of hyperphosphorylated tau protein (Wang and Mandelkow, 2016). Numerous observations suggest a close association between ApoE4 and tau pathology with respect to neurodegeneration, while initial histopathological examinations have corroborated a link between ApoE4 protein and NFTs in AD brains (Huang et al., 2001; Rohn et al., 2012; Brecht et al., 2004). Prior research has shown that ApoE3 binds to tau more effectively than ApoE4 at regions responsible for aggregation into pathogenic NFTs. This suggests that ApoE3 may prevent tau phosphorylation and the formation of NFTs (Strittmatter et al., 1994). Studies have also demonstrated that mice expressing ApoE4 in tauopathy models exhibit more severe tau-induced neurodegeneration compared to other isoforms, while mice lacking ApoE are protected from this effect (Shi et al., 2017). Furthermore, eliminating ApoE4 derived from astrocytes has been found to offer protection against tau-driven neurodegeneration (Wang et al., 2021). In addition, ApoE4 prompts tau-mediated neurotoxicity through its interaction with the vesicular monoamine transporter 2 (VMAT2), thereby impeding the transport of neurotransmitters into synaptic vesicles and ultimately leading to the degeneration of the locus coeruleus (Kang et al., 2021). Moreover, a recent study has indicated that reducing ApoE4 levels using antisense oligonucleotides in P301S/ApoE4 mice yields protective effects against tau pathology (Litvinchuk et al., 2021). Another study has demonstrated that ApoE4 exerts inhibitory effects on the Wnt signaling pathway by modulating LRP5/6 receptors, thereby leading to elevated GSK3 activity and facilitating tau phosphorylation (Caruso et al., 2006). In addition, ApoE4 has been shown to induce higher levels of tau phosphorylation in the presence of Aβ oligomers when compared to ApoE2 and ApoE3 (Hou et al., 2020). A recent genome-wide association study (GWAS) has suggested that ApoE2 distinctly attenuates the influence of the classical complement cascade on protein phosphatase 2A (PP2A) activity, a pivotal tau phosphatase in the human brain. This finding implies that ApoE2 may offer protective effects against the risk of AD in contrast to ApoE4 (Jun et al., 2022). In contrast, the use of PET imaging in a human study revealed that individuals carrying the ApoE4 gene variant show significantly higher levels of tau deposition, even when there are no Aβ plaques present. Studies using human iPSC-derived cell types have indicated that the presence of neuronal ApoE4 leads to more extensive tau phosphorylation and cell death compared to ApoE3 (Therriault et al., 2020). Data from human iPSC-derived cell types have shown that neuronal ApoE4 promotes tau phosphorylation and cell death to a greater extent than ApoE3 (Wadhwani et al., 2019). Additionally, examination of postmortem human brains has shown that individuals with two copies of the *ApoEε4* allele have higher levels of tau aggregates when Aβ is also present, in comparison to those with only one or no *ApoEε4* alleles (Tiraboschi et al., 2004). However, this association is not observed in brains without Aβ (Farfel et al., 2016).

### 3.3 ApoE and synaptic dysfunction and neuroinflammation

Synaptic dysfunction represents an early pathological hallmark of AD, preceding neurodegeneration and memory impairment, thereby culminating in cognitive decline and subsequent memory loss (Tzioras et al., 2023). Increasing evidence indicates that ApoE isoforms modulate synaptic function in an isoform-specific manner. Specifically, the ApoE3 isoform stimulates neurite outgrowth in both primary embryonic and adult cortical neurons and enhances neuronal sprouting within a developing organotypic hippocampal slice system (Nathan et al., 1994; Teter et al., 1999). In contrast, studies have demonstrated that ApoE4 exacerbates detrimental impacts on neurite outgrowth (Teter et al., 2002; Kim et al., 2014). Transgenic mouse models featuring neuronal overexpression of ApoE4 exhibited deficiencies in learning and vertical exploratory activity relative to those with neuronal overexpression of ApoE3 (Raber et al., 2000). A recent human study revealed that carriers of the *ApoEε4* allele manifested significant synaptic loss in the medial temporal lobe compared to non-carriers, indicating the adverse effects of ApoE4 on synaptic function (He et al., 2024). Although ApoE2 has not been extensively studied, a particular study provided evidence that ApoE2 mitigated dendritic spine loss in the hippocampus of young APP transgenic mice (Lanz et al., 2003). Conversely, ApoE4 has been found to exacerbate Aβ plaque and tau burden, leading to heightened neuronal injury and synaptic dysfunction, which are closely linked with neuroinflammation. This observation implies that ApoE-mediated neuroinflammation may exacerbate tau and Aβ aggregation, as well as neurodegeneration (Yamazaki et al., 2019; Zhang et al., 2023; Parhizkar and Holtzman, 2022).

A substantial body of literature suggests that ApoE is implicated in neuroinflammation, a pivotal characteristic of AD pathology. Microglia, frequently observed surrounding plaques in post-mortem brain tissue from individuals with AD, assume a central role in the immune response, as evidenced by pronounced reactive microgliosis (Song and Colonna, 2018). Interestingly, several studies have indicated that ApoE-null mice display a diminished microglial response to plaques, implying a potential regulatory role of ApoE in the microglial response to amyloid plaques (Ulrich et al., 2018; Wang et al., 2021; Krasemann et al., 2017). A recent study has reported that ApoE3 exhibits a higher capacity to stimulate a microglial response to injected Aβ compared to ApoE4. This effect may be mediated through the TREM2, which serves as an endogenous ligand of ApoE (Fitz et al., 2021). Notably, because of reduced lipidation and diminished affinity of ApoE4 for TREM2, ApoE4 has the potential to impede homeostatic microglial functions in comparison to other isoforms. This may lead to an acceleration of neuronal damage and disease progression (Fernandez et al., 2019). The targeting of ApoE-related neuroinflammation could represent a promising approach for the development of therapeutics for AD.

The decline in neuronal function represents a hallmark of advanced AD. The aggregation of Aβ and hyperphosphorylated tau, as well as neuroinflammation and synaptic dysfunction driven by ApoE, collectively contribute to progressive neurodegeneration (Yamazaki et al., 2019). Recent findings have demonstrated that the loss of ApoE function promotes neurodegeneration (Pankiewicz et al., 2021), highlighting the significant role of ApoE in neuronal loss. Additionally, a prior study has indicated that inhibiting endogenously synthesized cholesterol intensifies ApoE4-induced neuronal death (Michikawa and Yanagisawa, 1998). Therefore, gaining an understanding of the molecular mechanisms underpinning ApoE-mediated neuronal loss could potentially preserve neuronal function and decelerate disease progression.

Increased oxidative stress within neurons, attributed to an imbalance between free radicals and antioxidants, results in lipid peroxidation, protein oxidation, and DNA damage, thus contributing to neurodegeneration in AD (Erekat, 2022). Research indicates a direct correlation between the degree of oxidative damage in the AD brain and the ApoE allele, with the order of potency being ApoE2 < ApoE3 < ApoE4 (Dose et al., 2016; Butterfield and Mattson, 2020). Furthermore, a study demonstrated that AD patients carrying the *ApoEε4* allele display the most significant elevation of thiobarbituric acid reactive substances (TBARS) signals in the hippocampus, indicating heightened lipid peroxidation, a characteristic feature of oxidative stress (Ramassamy et al., 2000). Mice lacking ApoE4, when exposed to oxidative stress, showed elevated glutathione and other antioxidants in the brain; however, in ApoE-null mice, the increased glutathione levels did not reduce the oxidative stress, supporting the hypothesis that ApoE deficiency is associated with oxidative impairment in the brain (Butterfield and Mattson, 2020; Shea et al., 2002).

BBB is a critical component for maintaining the brain’s microenvironment. Research has demonstrated that the compromise of the BBB serves as a biomarker of cognitive impairment in humans, particularly in the early stages of AD (Nation et al., 2019). BBB dysfunction leads to heightened neuroinflammation and exacerbates the pathology of AD (Brandl and Reindl, 2023). A prior study noted the impairment of tight junction (TJ) barrier function when the BBB was reconstituted with primary astrocytes from ApoE4-KI mice (ApoE4-BBB model) (Nishitsuji et al., 2011). A recent investigation has indicated that the ApoE4 variant, identified as the principal susceptibility gene for AD, precipitates dysfunction of the BBB and exacerbates cognitive impairment (Montagne et al., 2020). This finding suggests that the mitigation of ApoE4-associated cognitive decline, irrespective of AD pathology, may represent a viable therapeutic objective for individuals carrying the *ApoEε4* allele.

Deregulated apoptosis, commonly known as programmed cell death, is recognized as a contributory element in neurodegenerative disorders, notably AD. Studies have evidenced that individuals with AD carrying the *ApoE ε4/ε4* allele demonstrate heightened apoptosis and compromised synaptic integrity, thereby aggravating the loss of synapses and neurodegeneration (Zhao et al., 2020). Consequently, the modulation of ApoE represents a potential therapeutic strategy for addressing apoptosis and programmed cell death.

### 3.4 ApoE and organelle dysfunction

Endoplasmic reticulum (ER) stress is a cellular response to the accumulation of misfolded proteins in the ER. The involvement of ApoE in ER stress has been associated with the pathogenesis of AD. One study has revealed that domain interaction in ApoE4 induces ER stress and impairs astrocyte function in Arg-61 ApoE mice, a gene-targeted mouse model specific for domain interaction (Zhong et al., 2009). Another study has demonstrated that ApoE4 expression leads to macrophage dysfunction and promotes apoptosis through the induction of ER stress (Cash et al., 2012). The multifaceted impact of ApoE on cellular physiology is highlighted by its role in ER stress.

Mitochondrial dysfunction assumes a prominent role in neurodegenerative diseases, notably AD. Impaired mitochondria can evoke oxidative stress and energy deficits, culminating in neuronal damage, inflammation, accumulation of plaque-forming Aβ peptides, and cognitive decline (Qin et al., 2020). Notably, evidence suggests that mitochondrial dysfunction serves as a regulator of ApoE-dependent cellular processes in both healthy and pathological states by exerting influence on ApoE expression and secretion (Wynne et al., 2023). Investigations have revealed that the ApoE4 (1–272) fragment associates with mitochondrial complexes, thereby instigating neurodegeneration through its impact on neuronal mitochondrial function (Nakamura et al., 2009). Moreover, recent findings demonstrate a correlation between the *ApoEε4* allele and compromised mitochondrial structure and function, oxidative stress, and synaptic integrity in the human brain, indicative of the potential for ApoE4 to precipitate neuronal damage (Yin et al., 2020).

ApoE facilitates the clearance of Aβ in a manner that is dependent on its isoform, with ApoE3 being more effective than ApoE4, by activating phagocytosis, which is a critical process for removing damaged organelles and protein aggregates (Koistinaho et al., 2004; Kanekiyo et al., 2013). A recent study has demonstrated that the expression of ApoE4 is associated with impaired autophagy and mitophagy in astrocytes (Eran and Ronit, 2022). This suggests that dysregulated autophagy may contribute to the accumulation of toxic Aβ aggregates, further exacerbating neurodegeneration in AD.

## 4 Novel functions of ApoE

Recent advances in the understanding of ApoE have revealed its protective role against PS mutation-induced FAD and its involvement in the pathology of AD driven by ApoE. Moreover, the interactions between ApoE and PS, and the inhibitory effect of ApoE4 on *γ*-secretase activity have been elucidated. These discoveries suggest a common underlying mechanism shared between FAD and SAD. Furthermore, recent studies have uncovered the impact of ApoE isoforms on the expression of APP and the modulation of Aβ secretion, as well as the influence of gut microbiota on ApoE function.

### 4.1 ApoE and its protective mutation

**Figure 3:**
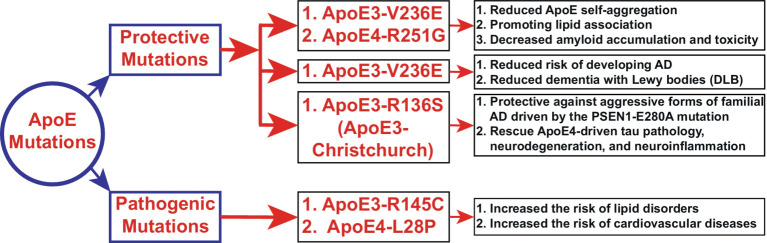
Novel functions of ApoE mutations (Section 4.1). The schematic diagram depicts the newly identified functions of ApoE3 and ApoE4 mutations. Polymorphism in the ApoE gene is the most significant risk factor for AD and other neurological disorders. Here, we elucidate the novel regulatory, protective, and pathogenic functions of ApoE mutations in AD and other neurodegenerative diseases. ApoE4 exerts toxic effects that are three to four times greater than those of ApoE3 in late-onset sporadic AD, leading to enhanced Aβ accumulation. However, the ApoE4-R251G mutation decreases amyloid accumulation and toxicity. On the other hand, ApoE3-R145C (ApoE3-Philadelphia) mutations have been associated with lipid disorders and cardiovascular diseases. Notably, most of the mutations shown here exert protective effects, suggesting that genetic mutations in ApoE3 or ApoE4 could be potential therapeutic strategies.

Despite the heightened risk of AD onset by three to four times associated with the presence of the ApoE4 gene, certain protective mutations have been elucidated. Specifically, both the ApoE4-R251G mutation and a variant of ApoE3-V236E, known as the Jacksonville mutation, demonstrate protective attributes. These variants are linked to mitigated ApoE self-aggregation, facilitating lipid association and potentially reducing amyloid accumulation and toxicity (Figure 3) (Le Guen et al., 2022; Bu, 2022; Sepulveda-Falla, 2023). Additionally, the ApoE3-V236E variant is correlated with a decreased susceptibility to AD and dementia with Lewy bodies (DLB) (Figure 3) (Liu et al., 2021; Medway et al., 2014). Conversely, mutations such as L28P in ApoE4 (ApoE4-Freiburg) or ApoE3-R145C (ApoE3-Philadelphia) have been associated with an elevated risk of lipid disorders and cardiovascular diseases (Figure 3) (Orth et al., 1999; Abou Ziki et al., 2014). Furthermore, individuals homozygous for the ApoE3-Ch have exhibited resistance against aggressive forms of FAD associated with the *PSEN1*-E280A mutation (Figure 3) (Arboleda-Velasquez et al., 2019). Recent research has indicated that homozygosity for the ApoE3-R136S mutation can mitigate ApoE4-driven tau pathology, neurodegeneration, and neuroinflammation in mouse models of tauopathy and human induced pluripotent stem cell (iPSC)-derived neuron models (Figure 3). In contrast, heterozygous carriers demonstrate partial protection against neurodegeneration and neuroinflammation but not against tau pathology (Nelson et al., 2023).

### 4.2 ApoE, APP, and Aβ production

**Figure 4:**
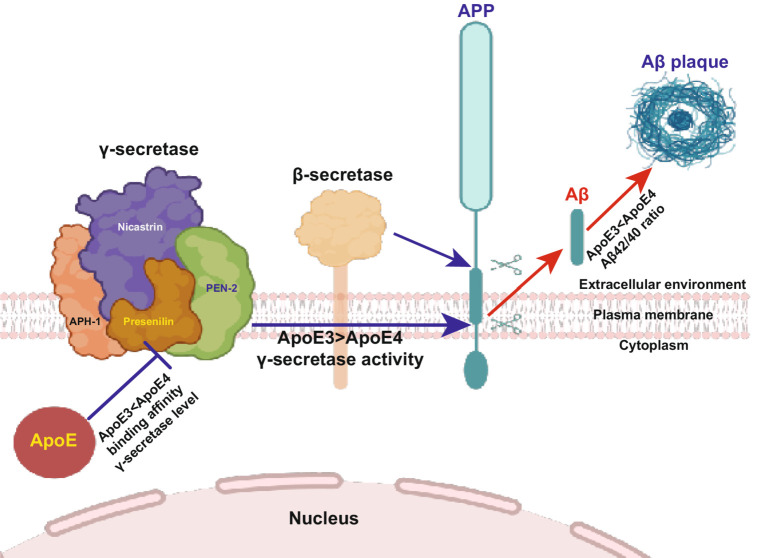
Higher binding affinity of ApoE4 to the γ-secretase complex inhibits γ-secretase activity (Section 4.2). Familial mutations in PS lead to impaired γ-secretase activity and altered Aβ production, resulting in a substantially increased Aβ42/Aβ40 ratio. Here, we illustrate the novel functions of ApoE in Aβ production. This finding elucidates the involvement of ApoE in the formation and activity of the γ-secretase complex. ApoE4 suppresses γ-secretase activity and increases the Aβ42/Aβ40 ratio compared to ApoE3, suggesting that Aβ production and γ-secretase activity are regulated by ApoE in an isoform-dependent manner. This study reveals a novel connection between ApoE and PS, the most important causative molecules in sporadic and familial Alzheimer’s disease, respectively. The diagram is sourced from Sun et al. (2023).

The generation of the Aβ peptide from APP represents a critical event in the pathogenesis of AD. Recent research indicates the involvement of ApoE in the processing of APP and subsequent Aβ generation. Specifically, ApoE, secreted by glial cells, has been shown to stimulate neuronal Aβ production, with ApoE4 exhibiting a greater potency in comparison to ApoE3 and ApoE2 (Huang et al., 2017). Furthermore, investigations involving human neurons derived from induced pluripotent stem cells (iPSCs) carrying the *ApoEε4* allele have revealed elevated secretion levels of Aβ when compared to those harboring the *ApoEε3* allele. This disparity in Aβ secretion may be attributed to augmented APP transcription or processing (Lin et al., 2018; Wang et al., 2018). Our recent investigation has revealed that intracellular ApoE4 significantly hinders Aβ40 production more than Aβ42 production, resulting in an elevated Aβ42/Aβ40 ratio due to its interaction with the γ-secretase complex. This discovery proposes a novel pathway through which intracellular ApoE4 contributes to the progression of SAD by impeding γ-secretase activity (Figure 4) (Sun et al., 2023).

### 4.3 ApoE and microbiota

**Figure 5:**
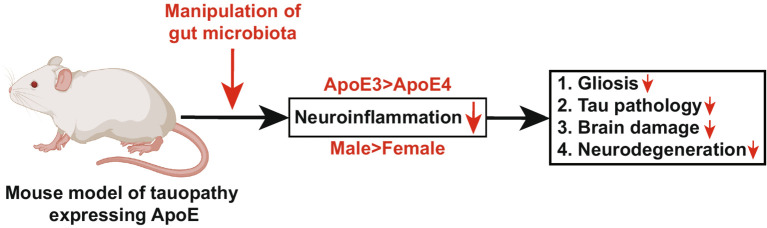
Manipulation of gut microbiota in a mouse model of tauopathy expressing human ApoE attenuates neurodegeneration (Section 4.3). This diagram illustrates the role of ApoE and gut microbiota in controlling neurodegeneration. The progression of tau-mediated neurodegeneration is regulated by ApoE-driven neuroinflammation, and emerging evidence suggests that gut microbiota regulates neuroinflammation in an ApoE-genotype-dependent manner. In this study, we demonstrated that a genetically modified mouse model of tauopathy expressing human ApoE isoforms, manipulated by gut microbiota, exhibited reduced gliosis, tau pathology, brain damage, and neurodegeneration in a sex- and ApoE-isoform-dependent manner. This finding reveals a novel mechanism linking gut microbiota, ApoE-mediated neuroinflammation, and tau-driven neurodegeneration. The diagram is sourced from Seo et al. (2023).

ApoE-mediated neuroinflammation is understood to play a pivotal role in tau-mediated neurodegeneration, and there is evidence that the gut microbiota exerts control over neuroinflammation in an ApoE genotype-dependent manner. A study conducted on a genetically modified mouse model of tauopathy, expressing human ApoE isoforms, revealed a decrease in gliosis, tau pathology, and neurodegeneration. This reduction was influenced by both sex (male > female) and specific ApoE isoforms (ApoE3 > ApoE4). These findings unveiled a substantial interplay between microbiota composition, neuroinflammation, and tau-mediated neurodegeneration (Figure 5) (Seo et al., 2023). Several studies have underscored the increasing significance of the gut microbiome in impacting AD pathology through its influence on metabolism and immune function. Notably, insulin supplementation has been demonstrated to enhance metabolism, resulting in heightened levels of beneficial microbiota and reduced neuroinflammation, particularly in ApoE4 mouse models (Hoffman et al., 2019). Moreover, the presence of the *ApoEε4* allele has been associated with modified levels of gut microbiota in AD patients. Consequently, directing interventions toward the microbiota may constitute an efficacious therapeutic strategy for AD individuals bearing the *ApoEε4* allele (Hou et al., 2021). These collective findings elucidate a multifaceted interplay between the ApoE genotype, gut microbiota, neuroinflammation, and AD pathology, consequently delineating potential therapeutic trajectories targeting microbiota-related mechanisms in AD.

## 5 Relationship of ApoE with other neurodegenerative diseases

Numerous studies have demonstrated the multifaceted impact of ApoE alleles on neurodegenerative diseases. Our research delves into the diverse roles of ApoE across diseases such as PD, ALS, FTLD, HD, VD and MS, offering new insights into its multifaceted significance.

### 5.1 ApoE and Parkinson’s disease (PD)

PD is the second most prevalent neurodegenerative disorders, characterized by the accumulation of protein aggregates known as Lewy Bodies (LBs) within brain cells. These LBs primarily consist of *α*-synuclein along with other proteins and lipids, making their composition highly complex (Calabresi et al., 2023; Burré et al., 2018; Vázquez-Vélez and Zoghbi, 2021; Emamzadeh, 2017). It is hypothesized that cholesterol-rich domains, such as lipid rafts, may act as focal points for α-synuclein aggregation. Additionally, cholesterol is believed to regulate α-synuclein binding to structures resembling synaptic vesicles, potentially initiating aggregation processes (Sarchione et al., 2021). Recent research has uncovered elevated levels of ApoE, a lipid transport protein, in the cerebrospinal fluid of individuals in early stages of PD. This finding suggests that α-synuclein might facilitate its movement between neurons by interacting with ApoE, which is involved in lipid transport mechanisms (Paslawski et al., 2019). Moreover, studies indicate that high levels of fatty acids can induce α-synuclein aggregation, contributing to LB formation (Erskine et al., 2021). In the context of fatty acids (FAs), harmful forms can be generated in hyperactive neurons and transferred to astrocyte lipid droplets via ApoE-positive lipid particles. Within astrocytes, these FAs undergo mitochondrial beta-oxidation for detoxification (Ioannou et al., 2019). However, pathogenic α-synuclein has been implicated in impairing astrocyte mitochondria function (Braidy et al., 2013). This dysfunction can disrupt FA metabolism in astrocytes, potentially leading to neuronal damage from toxic FAs and exacerbating the progression of PD (Braidy et al., 2013). In summary, disruptions in FA metabolism mediated by astrocyte mitochondria dysfunction may contribute to the pathogenesis and progression of PD, highlighting potential targets for therapeutic interventions.

Recent studies have highlighted the role of ApoE variants in PD and related cognitive decline. Cognitive decline appears to progress more rapidly in PD patients carrying ApoE4 variants compared to single variant carriers (Jo et al., 2021). *In vitro* studies investigating different ApoE isoforms have shown that ApoE4 increases α-synuclein aggregation more than other isoforms, which may exacerbate disease progression (Emamzadeh, 2016). Additionally, research conducted in Spain indicated that the *ApoEε4* allele is more prevalent in familial PD but less common in sporadic PD compared to control groups, suggesting a potential risk association in individuals with specific genetic backgrounds (Blázquez et al., 2006). However, other studies have reported conflicting findings regarding ApoE genotypes and PD risk (Federoff et al., 2012; Multhammer et al., 2014).

Clinical and genetic evidence points to variants in the ApoE gene as significant risk factors for LBD and the onset of dementia in PD patients (Kaivola et al., 2022; Pankratz et al., 2006). Specifically, ApoE4 variants are associated with more severe LB pathology independent of AD pathology (Dickson et al., 2018; van Steenoven et al., 2020). ApoE4 also increases the risk of PD-related dementia and may lead to earlier symptom onset. Animal studies have further demonstrated that ApoE4 influences α-synuclein pathology and exacerbates its detrimental effects, suggesting a role in impairing α-synuclein clearance and promoting neuroinflammation (Zhao et al., 2020; Davis et al., 2020). These processes could contribute to neuronal dysfunction and degeneration in LBD. The relationship between ApoE and PD is complex and likely involves interactions with other genetic and environmental factors. Further research is crucial to elucidate the precise mechanisms underlying the association between ApoE variants and PD susceptibility and progression.

### 5.2 ApoE and amyotrophic lateral sclerosis (ALS)

ALS is a devastating neurodegenerative disease that affects upper and lower motor neurons in the brain and spinal cord, leading to progressive muscle weakness, stiffness, and eventual paralysis (Mead et al., 2023; Longinetti and Fang, 2019; Kim et al., 2020). ALS can occur sporadically or in familial forms, with mutations in genes like superoxide-dismutase1 (SOD1) and TDP-43 being common causes that result in nerve cell degeneration and death (Jeon et al., 2019; Brown et al., 2021; Tziortzouda et al., 2021). The ApoE gene, crucial for lipid transport and metabolism, is well-established as a genetic risk factor for AD, with the *ApoEε4* allele notably linked to increased susceptibility (Fernández-Calle et al., 2022; Verghese et al., 2011). However, its role in ALS remains less clear. Studies exploring the *ApoEε4* allele association with ALS risk or disease progression have produced inconsistent findings. In ALS, TDP43 pathology is prevalent in nearly all sporadic cases, affecting 90 to 95% of patients (Oiwa et al., 2023). Recent research indicates a potential interplay between ApoE genotype and TDP-43 pathology in ALS. Overexpression of TDP-43 can lead to motor deficits, neuronal loss, and gliosis in the motor cortex, with these effects being most severe in ApoE2 mice. Additionally, observations from human postmortem brain samples suggest an association between the ApoE*ε2* allele and more pronounced TDP-43 pathology, implying that genetic factors such as ApoE genotype may influence the progression and severity of ALS pathology (Meneses et al., 2023).

Recent research has indicated a potential link between mitochondrial dysfunction and ApoE expression and secretion in ALS (Wynne et al., 2023). This suggests that mitochondrial impairment may influence ApoE levels, which could in turn contribute to the neurodegenerative processes observed in ALS. Moreover, lipid dysregulation or lipid cacostasis is one of the pathological hallmarks of ALS, contributing to neurodegeneration (González De Aguilar, 2019; Dodge et al., 2020). In addition to mitochondrial dysfunction, lipid dysregulation is another hallmark of ALS pathology (Abdel-Khalik et al., 2017). Studies have reported defective cholesterol metabolism in the brains of ALS patients and abnormal lipid droplet accumulation in astrocytes, particularly in regions like the motor cortex and spinal cord (Chaves-Filho et al., 2019). These lipidomic findings underscore the importance of lipid homeostasis in ALS pathophysiology and suggest that disruptions in lipid metabolism may exacerbate neurodegeneration. Despite these insights, the precise mechanisms through which ApoE and lipid dysregulation contribute to ALS remain complex and multifaceted. Ongoing research continues to investigate these genetic factors and their interactions with mitochondrial function and lipid metabolism in ALS, aiming to uncover novel therapeutic targets for this devastating disease.

### 5.3 ApoE and frontotemporal lobar degeneration (FTLD)

FTLD comprises a spectrum of neurodegenerative disorders primarily affecting the frontal and temporal lobes, resulting in progressive changes in behavior, personality, language, and motor function (Mann and Snowden, 2017; Grossman et al., 2023). These disorders are characterized by the accumulation of different protein aggregates, including tau, TDP-43, or fused in sarcoma (FUS) proteins, depending on the specific subtype of FTLD (Riku et al., 2022; Irwin et al., 2015). The impact of ApoE polymorphism on FTLD has been a subject of scientific investigation, but findings have been inconsistent and contradictory across different populations. For instance, in the Dutch population, *ApoEε4* allele has been associated with an increased risk of FTLD (Stevens et al., 1997). Conversely, studies in German patients have reported a negative association between ApoE polymorphism and FTLD risk (Riemenschneider et al., 2002). A comprehensive meta-analysis encompassing Caucasian and Asian populations concluded that *ApoEε4* allele serves as a genetic risk factor for FTLD (Su et al., 2017). However, GWAS on FTLD have not consistently validated a positive association with the ApoE gene (Ferrari et al., 2014; Girard and Rouleau, 2014). Unlike its well-established role in AD, where *ApoEε4* allele significantly increases risk, the influence of *ApoEε4* allele on FTLD risk appears to be less pronounced. Research indicates that the presence of the *ApoEε4* allele does not substantially elevate the risk of FTLD as it does with AD. This discrepancy underscores the complexity of genetic and molecular factors underlying FTLD and necessitates further investigation to elucidate the precise relationship between ApoE and FTLD pathogenesis.

### 5.4 ApoE and Huntington’s disease (HD)

HD is a progressive neurodegenerative disorder caused by a genetic mutation involving the huntingtin gene (HTT), characterized by motor dysfunction, cognitive decline, and psychiatric symptoms (Margolis et al., 2001; van der Burg et al., 2009). Research in both human subjects and animal models has highlighted significant alterations in cholesterol metabolism as a pivotal early event in HD pathogenesis (Trushina et al., 2006; del Toro et al., 2010; Boussicault et al., 2016; Kacher et al., 2022). Astrocytes, specialized glial cells in the brain, play a crucial role in maintaining cholesterol homeostasis, which becomes disrupted in HD. This disruption manifests through the decreased expression of genes involved in cholesterol biosynthesis and transport, leading to reduced levels and impaired secretion of ApoE lipoproteins (Valenza et al., 2010; Valenza et al., 2015). ApoE is vital for transporting lipids and aiding in repair processes within the brain. A deficiency in ApoE exacerbates the impact of reduced cholesterol availability on neurons, potentially contributing to the observed neuronal dysfunction and degeneration in HD. Additionally, HD models display a decrease in ApoE expression (Kacher et al., 2022) and mitochondrial genes (Intihar et al., 2019), indicating a potential link to impaired cholesterol metabolism and mitochondrial function. ApoE proteins play critical roles in cellular pathways associated with mitochondrial function (Lee et al., 2023). Disruptions in these interactions, particularly in the context of HD, could exacerbate mitochondrial dysfunction and contribute to disease progression. Therefore, there is growing interest in exploring strategies to modify ApoE expression or function as a potential therapeutic approach to mitigate mitochondrial dysfunction in HD. This could involve innovative techniques such as gene therapy, small molecule interventions targeting ApoE pathways, or other approaches aimed at restoring mitochondrial health in affected cells.

### 5.5 ApoE and vascular dementia (VD)

VD is a prevalent cause of dementia after AD, resulting from brain damage due to compromised blood flow. This condition accounts for approximately 15% of dementia cases (O'Brien and Thomas, 2015). VD shares a 20–30% prevalence of dementia along with other pathologies, manifesting in impaired reasoning, planning, judgment, memory, and other cognitive processes (Jellinger, 2013). Studies have identified similar pathological features between VD and AD, inclusive of neurofibrillary tangles, amyloid plaques, white-matter lesions, and CAA. ApoE represents a significant risk factor for VD, with meta-analyses demonstrating heightened susceptibility to VD in individuals with the *ApoEε4* allele compared to those with the *ApoEε3* allele (Yin et al., 2012). Recent investigations suggest that ApoE4 exacerbates advanced-stage vascular and neurodegenerative disorders in aged AD mice, leading to BBB breakdown, diminished cerebral blood flow, neuronal loss, and behavioral deficits independently of Aβ (Montagne et al., 2021). Thus, the presence of ApoE polymorphism in VD patients may establish a potential connection between VD and AD and elevate the risk for VD.

### 5.6 ApoE and multiple sclerosis (MS)

MS represents a prevalent autoimmune disorder affecting the central nervous system within the young adult population aged 18–40 years (Jakimovski et al., 2024). Recent pathophysiological insights underscore the significance of the genetic linkage between the chromosome 19q13 region and MS (Sawcer et al., 2002; Giau et al., 2015). The association between ApoE genotype and MS susceptibility remains ambiguous, although a study has suggested that both the *ApoEε2* and *ApoEε4* alleles may exert substantial effects on MS susceptibility (Lill et al., 2012). Furthermore, investigations have proposed that the presence of the *ApoEε4* allele in MS patients may contribute to disease progression, leading to heightened brain damage, exacerbated cognitive dysfunction, and augmented disease severity (Parmenter et al., 2006; Shi et al., 2008; Oliveri et al., 1999; Sahebari et al., 2023). Additionally, it has been reported that MS patients carrying the *ApoEε4* allele demonstrate deficits in verbal memory (Shi et al., 2008; Koutsis et al., 2007). Consequently, the influence of ApoE4 on MS pathogenesis appears predominantly adverse.

## 6 ApoE-targeted therapeutic strategies, treatment, and research progress

Extensive research on AD has spurred inquiries into therapeutic approaches targeting amyloid aggregates, a defining feature of the disease. Notable avenues of interest involve pharmacotherapies such as aducanumab and lecanemab, which possess a specific affinity for amyloid plaques. While these pharmaceuticals have been subjected to clinical trials, further investigation is imperative to ascertain their long-term safety and efficacy (Raulin et al., 2022; Sperling et al., 2011; Sevigny et al., 2016). Some studies demonstrated that ApoE4 homozygotes have a very high risk for Amyloid-related imaging abnormalities (ARIA) with both aducanumab and lecanemab and many providers do not administer these medications in ApoE4 homozygote carriers for this reason (Sperling et al., 2012; VandeVrede et al., 2020). Additionally, it remains to be explored whether the ApoE genotype influences the safety and efficacy of these therapies. Current therapeutic strategies for AD also concentrate on modulating ApoE levels and lipidation status, targeting ApoE’s structural attributes, and its interactions with A*β*. Recognized for its pivotal role in lipid transportation and preservation of synaptic homeostasis, ApoE presents itself as a viable therapeutic target for AD. Evidence suggests that augmenting ApoE levels or enhancing its lipidation could prove advantageous in AD treatment. Notably, in murine models of amyloid pathology, ligands of liver X receptor (LXR) and retinoid X receptor (RXR) have demonstrated the capability to enhance the transcription and secretion of all ApoE isoforms, thereby mitigating Aβ deposition and reinstating cognitive function (Yamazaki et al., 2019; Fernández-Calle et al., 2022). Additionally, ABCA1, which is transcriptionally regulated by LXR and is crucial for cholesterol efflux and HDL formation, is affected by the self-aggregation of ApoE4, which reduces ABCA1 membrane recycling and leads to decreased lipidation of ApoE4 particles (Fernández-Calle et al., 2022; Rawat et al., 2019).

Furthermore, in a mouse model of amyloid pathology, AAV-mediated human ApoE gene expression revealed that the presence of the *ApoEε4* allele exacerbated synaptic loss and Aβ deposition, while the *ApoEε2* allele expression in the same model led to a reduction in brain Aβ levels (Yamazaki et al., 2019; Hudry et al., 2013). Deletion of the gene encoding ABCA1, responsible for lipidating ApoE in the CNS, resulted in an increase in Aβ deposition (Wahrle et al., 2005) Conversely, overexpression of ABCA1 resulted in a decrease in Aβ deposition (Wahrle et al., 2008) in amyloid mouse models, suggesting that increasing ApoE lipidation can reduce Aβ pathology. Various studies have demonstrated that ApoE immunotherapy targeting ApoE-mediated plaque formation, using anti-mouse ApoE antibody HJ6.3 and/or anti-human ApoE antibody HAE-4, induced a significant reduction in Aβ deposition in a mouse model of amyloid pathology (Kim et al., 2012; Liao et al., 2018). Additionally, treatment with peptides bearing characteristics similar to native ApoE has shown promise in reducing Aβ deposition (Vitek et al., 2012; Handattu et al., 2013), tau hyperphosphorylation (Ghosal et al., 2013), and glial activation in mouse models of amyloid pathology. However, the potential impact of these findings on human subjects requires further exploration (Yamazaki et al., 2019).

The targeted modification of ApoE4 structural properties using PH002, an inhibitor of intracellular domain interaction of ApoE4, has demonstrated a reduction in ApoE4 fragmentation and its associated effects on Aβ production, tau phosphorylation, and GABAergic neuron degeneration in human iPSC-derived neurons (Wang et al., 2018; Mahley and Huang, 2012b). ApoE4, due to its pathological conformation compared to ApoE3 and ApoE2, is considered the primary risk factor for SAD. Consequently, the conversion of ApoE4 to ApoE3 or ApoE2 represents a promising therapeutic approach for AD treatment. Studies have indicated that editing the *ApoEε4* allele to the *ApoEε3* allele via gene-editing technologies leads to a reduction in ApoE fragmentation, Aβ production, tau phosphorylation, and GABAergic neuron degeneration in iPSC-derived neurons, suggesting that the detrimental effects of ApoE4 could be alleviated through gene editing (Yamazaki et al., 2019; Wang et al., 2018). Inhibition of the interaction between ApoE and Aβ demonstrates potential benefits in mitigating Aβ pathology. Administration of the synthetic peptide Aβ12–28P, structurally akin to the ApoE binding site on full-length Aβ, has exhibited a reduction in insoluble tau accumulation in AD mouse models (Liu et al., 2014). Additionally, it has led to diminished brain Aβ buildup, coupled with concurrent ApoE co-deposition within Aβ plaques and neuritic degeneration in amyloid mouse models characterized by ApoE2-TR and ApoE4-TR backgrounds (Pankiewicz et al., 2014).

The targeting of ApoE receptors presents a promising therapeutic approach for mitigating Aβ pathology. It is firmly established that the clearance of Aβ in the brain is, in part, facilitated by ApoE receptors, namely LDLR and LRP1 (Bu, 2009; Kanekiyo et al., 2014; Zhao et al., 2018). Research has demonstrated that the upregulation of LDLR augments Aβ clearance, leading to a significant reduction in Aβ deposition in murine models of amyloid pathology (Kim et al., 2009). Moreover, the clinical exploration of fluvastatin, a compound for AD therapy, suggests its potential in diminishing Aβ deposition and/or enhancing Aβ clearance, potentially through the augmentation of LRP1 expression (Shinohara et al., 2010). Nevertheless, further investigation is imperative to fully elucidate the role of ApoE in mediating Aβ clearance via ApoE receptors.

Recently, a rare ApoE variant, ApoE3-Ch, was found to protect against both early-onset AD and in late onset AD (Nelson et al., 2023). This variant has gained significant attention in the AD field, as its homozygosity was associated with remarkable resistance to a severe form of familial AD linked to the *PSEN1-E280A* mutation (Arboleda-Velasquez et al., 2019). Individuals carrying two copies of this rare allele exhibited significantly reduced tau pathology and neurodegeneration, along with preserved glucose metabolism and cognitive function (Arboleda-Velasquez et al., 2019). Additionally, ApoE3-Ch/ApoE3-Ch astrocytes were shown to effectively mitigate tau propagation in iPSC-derived neurons (Murakami et al., 2024). Studies in a humanized APOE3-Ch knock-in mouse model, crossed with an Aβ plaque-depositing model, demonstrated that ApoE3-Ch alters microglial responses and suppresses A*β*-induced tau seeding and spreading (Chen et al., 2024). Furthermore, the homozygous ApoE4-R136S mutation (ApoE4-Ch) was shown to rescue ApoE4-driven tau pathology, neurodegeneration, and neuroinflammation in tauopathy mouse models and human iPSC-derived neurons carrying human ApoE4 with either homozygous or heterozygous R136S mutations in late-onset AD. While the heterozygous ApoE4-Ch mutation partially protected against ApoE4-driven neurodegeneration and neuroinflammation, it did not prevent tau pathology. Single-nucleus RNA sequencing revealed that the ApoE4-Ch mutation increased protective cell populations and reduced disease-associated populations in a gene dose-dependent manner (Nelson et al., 2023). These findings highlight the potential of the ApoE-Ch mutation to protect against ApoE4-driven AD pathologies, offering a promising target for therapeutic development against AD.

## 7 Conclusion

This review examines the correlation between the diverse functions of ApoE and its role in the development of various neurodegenerative conditions, including AD, PD, ALS, FTLD, HD, VD, and MS. The three primaries human ApoE polymorphisms, ApoE2, ApoE3, and ApoE4, exhibit differential effects on A*β* clearance and aggregation within the brain. Additionally, they play distinct roles in redistributing cholesterol and phospholipids to neurons through their interaction with cell-surface ApoE receptors. These polymorphisms also influence glucose metabolism, neuronal signaling, and neuroinflammation. ApoE is also pivotal in tau pathology, tau-mediated neurodegeneration, and the microglial response to AD-related pathologies. Recent investigations have shed light on novel functions of ApoE, notably its interaction with presenilin, the protective role of ApoE mutations against AD, and the ApoE4-driven tau pathology and neurodegeneration. Furthermore, ApoE isoforms, particularly ApoE4, demonstrate involvement in *α*-synuclein aggregation, TDP-43 and FUS-induced neurodegeneration, HTT gene regulation, MS susceptibility, and disruption of BBB. ApoE also governs vascular health, an area of significance given the strong association between vascular pathology and neurodegenerative diseases. Accordingly, the comprehensive study of ApoE and its multifaceted functions may unveil novel strategies for early disease risk identification. Emphasizing the link between ApoE and the risk of pathogenesis in various neurodegenerative diseases is imperative for human disease diagnosis, risk assessment, prevention, and treatment. Despite persistent challenges, ongoing ApoE research instills optimism for pioneering therapeutic interventions capable of attenuating or arresting the progression of neurodegenerative diseases.

## 8 Methods

Our team undertook an exhaustive literature review focusing on the multifunctionality of ApoE in neurodegenerative disorders. Employing systematic keyword searches and delving into highly referenced classical articles within databases such as PubMed and ScienceDirect allowed us to comprehensively cover recent research. Our primary objective was to select recent publications to acquire the most current insights into ApoE’s diverse roles in various neurodegenerative disorders. Prioritizing classical articles with substantial citations provided a robust theoretical basis for our review, facilitating a thorough exploration of ApoE’s involvement. Genetic variation data was sourced from AlzForum URL.^1^ This approach to literature review was designed to offer substantial support for our study, thus enriching the overall understanding of ApoE’s multifunctionality within the realm of neurodegenerative disorders.

## Footnotes

^1^ https://www.alzforum.org/mutations/apoe
